# Novel Alzheimer risk factor IQ motif containing protein K is abundantly expressed in the brain and is markedly increased in patients with Alzheimer’s disease

**DOI:** 10.3389/fncel.2022.954071

**Published:** 2022-07-19

**Authors:** Hongjie Wang, Dinesh Devadoss, Madhavan Nair, Hitendra S. Chand, Madepalli K. Lakshmana

**Affiliations:** ^1^Institute for Human Health and Disease Intervention (I-HEALTH), Department of Chemistry and Biochemistry, Center for Molecular Biology and Biotechnology, Florida Atlantic University, Jupiter, FL, United States; ^2^Department of Immunology and Nano-Medicine, Herbert Wertheim College of Medicine, Florida International University, Miami, FL, United States

**Keywords:** IQCK, Alzheimer’s disease, brain regions, neurons, microglia, oligodendrocytes, astrocytes, colocalization

## Abstract

Alzheimer’s disease (AD) is complex and highly heterogeneous. Less than 10% of AD cases are early-onset (EOAD) caused by autosomal dominantly inherited mutations in amyloid precursor protein (APP), presenilin 1 (PS1), or presenilin 2 (PS2), each of which can increase Aβ generation and, thus, amyloid plaques. The remaining 90% of cases of AD are late-onset (LOAD) or sporadic. Intense research efforts have led to identification of many genes that increase the risk of AD. An IQ motif containing protein K (IQCK) was recently identified by several investigators as an Alzheimer’s disease risk gene. However, how IQCK increases AD risk is completely unknown. Since IQCK is a novel gene, there is limited information on its physiological characterization. To understand its role in AD, it is first important to determine its subcellular localization, whether and where it is expressed in the brain, and what type of brain cells express the IQCK protein. Therefore, in this study, we show by immunocytochemical (ICC) staining that IQCK is expressed in both the nucleus and the cytoplasm of SH-SY5Y neuroblastoma cells as well as HeLa cells but not in either HMC3 microglial or CHO cells. By immunohistochemistry (IHC), we also show that IQCK is expressed in both mouse and human neurons, including neuronal processes *in vivo* in the mouse brain. IHC data also show that the IQCK protein is widely expressed throughout the mouse brain, although regional differences were noted. IQCK expression was highest in the brainstem (BS), followed by the cerebellum (CB) and the cortex (CX), and it was lowest in the hippocampus (HP). This finding was consistent with data from an immunoblot analysis of brain tissue homogenates. Interestingly, we found IQCK expression in neurons, astrocytes, and oligodendrocytes using cell-specific antibodies, but IQCK was not detected in microglial cells, consistent with negative *in vitro* results in HMC3 cells. Most importantly, we found that actin-normalized IQCK protein levels were increased by 2 folds in AD brains relative to normal control (NC) brains. Furthermore, the IQCK protein was found in amyloid plaques, suggesting that IQCK may play a pathogenic role in either Aβ generation or amyloid plaque deposition in AD.

## Introduction

Alzheimer’s disease (AD) is a chronic neurodegenerative disease clinically characterized by deterioration in learning, memory, and cognitive functions that ultimately affect behavior, speech, visuospatial orientation, and the motor system (Scheltens et al., 2021). AD is pathologically characterized by neuroinflammation, extensive neuronal loss, and accumulation of intracellular neurofibrillary tangles and extracellular amyloid plaques in the brain. AD is the most common neurodegenerative disease globally (Erkkinen et al., 2018) with roughly 35 million individuals affected (Prince et al., 2013). Throughout the world, the proportion of people aged 60 and older is growing faster (Zamudio-Rodríguez et al., 2021), and in the United States, by 2060, older persons are predicted to represent 25.8% of the population from the current 14.1% (Colby and Ortman, 2015). Since age is the greatest known risk factor for AD, roughly doubling every 5 years after the age of 65, shortly, there will be a significant increase in the incidence of AD. However, despite rigorous research efforts as reflected by recently completed hundreds of clinical trials, therapeutics targeting two hallmarks of AD, Aβ and tau, have mostly failed so far (Mehta et al., 2017; Congdon and Sigurdsson, 2018; van Dyck, 2018), with few exceptions targeting cholinergic and glutamatergic pathways that provide only symptomatic relief for a short time, indicating that novel pathways and molecular targets must be identified to bring disease-modifying therapy for AD.

Alzheimer’s disease has been suggested to have a strong genetic basis and has heritability estimates ranging from 60 to 80% (Gatz et al., 2006; Zusso et al., 2018). However, genetic variants in four well-established genes for early-onset AD (EOAD), namely, amyloid precursor protein (APP), presenilin (PS) 1 and PS2, the APoE4 allele, and the newly identified nine genetic risk factors for late-onset AD (LOAD), altogether account for less than half of this heritability (Kamboh et al., 2012; Blumenau et al., 2020). Therefore, additional risk genes need to be identified, characterized, and targeted for AD therapeutics. LOAD genes such as BIN1, CLU, CR1, PICALM, MS4A6A, ABCA7, EPHA1, CD33, and CD2AP were identified by genome-wide association study (GWAS) and were replicated by at least 2 independent GWASs, which also presented the strongest effect sizes after APOE (Buniello et al., 2019; Li et al., 2021). These novel genetic loci shed light on critical LOAD pathogenic pathways that include immune response (CD33, CR1, EPHA1, MSA4A cluster, CD2AP, and ABCA7), lipid metabolism (CLU and ABCA7), Aβ40–42 clearance (BIN1, CD33, PICALM, and ABCA7), and vesicle trafficking (BIN1 and PICALM). Genetic mechanisms that explain increased susceptibility to LOAD include polymorphisms in genetic sequence, mostly in intronic or intergenic regions (Naj et al., 2011; Keenan et al., 2012; Malik et al., 2013; Sassi et al., 2016), possibly modulating the susceptibility to disease in populations where they were analyzed. With three new GWAS studies published in 2018 and 2019, the number of genome-wide risk loci for AD increased to 40 (Marioni et al., 2018; Jansen et al., 2019; Kunkle et al., 2019).

Alzheimer’s disease is a multifactorial, heterogeneous, and enormously complex disorder. Thus, several other comorbidities are also known to be associated with AD. For example, midlife obesity confers increased risk of AD in later life, whereas late-life obesity is associated with decreased risk of AD (Whitmer et al., 2007; Beydoun et al., 2008; Chen et al., 2010). In an exploratory cross-disorder meta-analysis of the Genetic and Environmental Risk in Alzheimer’s Disease (GERAD) study for both obesity and AD, an IQ motif containing K gene (IQCK) was identified that showed the same direction of a risk allele for both AD and obesity (Hinney et al., 2014). IQCK as a risk locus in obesity was also confirmed by another independent study (Speliotes et al., 2010). Another study (Mattheisen et al., 2015) revealed a gene-level association of IQCK with obsessive-compulsive disorder (OCD), which is also known to increase the risk of developing AD (Chen et al., 2021). Importantly, a genetic meta-analysis of patients diagnosed with Alzheimer’s disease confirmed IQCK as a risk gene and ranked it at the top among other analyzed risk factors for AD (Kunkle et al., 2019). Using another rare variant (RV) quantitative non-parametric linkage (QNPL) method, which evaluates sharing of even minor alleles, recently, Zhao et al. (2020) confirmed IQCK’s role in AD risk in both *APOE* ε4-positive as well as negative family members. Thus, after the application of stringent quality control protocols, these multiple studies have confirmed the importance of the IQCK gene in increasing the risk of not only AD but also OCD and obesity. However, so far, nothing is known about IQCK gene function, protein localization in brain cells such as neurons, astrocytes, oligodendrocytes, and microglia, or regional distribution in brain regions. Therefore, here for the first time using several IQCK antibodies, we explored IQCK protein localization in multiple brain cell types using cell-specific markers by immunohistochemistry, in different brain regions of mice by immunoblotting, and finally compared IQCK protein expression in normal control subjects and with those of AD patient brains.

## Materials and methods

### Chemicals and antibodies

A protease inhibitor cocktail (cat # P8340), dithiothreitol (cat # D9779), and sodium orthovanadate (cat # 450243) were purchased from Sigma Aldrich (St. Louis, MO, United States). Microcystin-LR (cat# 475815) was purchased from Calbiochem-Millipore (Temecula, CA, United States). The Nonidet-P40 substitute (cat # M158) for lysis buffer preparation was obtained from Amresco (Solon, OH, United States). Syn-PER™ Synaptic Protein Extraction Reagent (cat # 87793) was purchased from Thermo Fisher Scientific (Waltham, MA, United States). USDA-certified fetal bovine serum (FBS) for cell cultures was purchased from BioFluid Technologies (cat # SKU: 100-500-Q). SuperSignal™ West Pico PLUS Chemiluminescent Substrate (cat # 34578) and PageRuler™ Prestained Protein Ladder, 10 to 180 kDa (cat # 26617) were purchased from Thermo Fisher Scientific. Polyclonal IQCK antibodies were purchased from BIOSS America (cat # BS-9023R), Proteintech (cat # 25740-1-AP), Novus Biologicals (cat # NBP156735), and Aviva Systems Biology (cat # ARP53134_P050). The Olig2 mouse monoclonal antibody was from Proteintech (cat # 66513-14g). GFAP Monoclonal Antibody (2.2B10) was purchased from Thermo Fisher Scientific (cat # 03-0300). Anti-NeuN Antibody, clone A60 (cat # MAB377), and a monoclonal anti-Iba1 antibody produced in mice (cat # SAB2702364) were obtained from Millipore Sigma (St. Louis, MO, United States). The Mouse 6E10 antibody for Aβ detection was purchased from Covance (cat # SIG-39320). The monoclonal anti-actin antibody deposited by Jim Jung-Ching Lin was purchased from the Development Studies Hybridoma Bank (DSHB), University of Iowa (Iowa City, IA, development studies hybridoma bank). Secondary antibodies such as peroxidase-conjugated AffiniPure goat anti-rabbit (code # 111-035-144) IgG (H + L) and goat anti-mouse (Code # 115-035-146) were purchased from Jackson ImmunoResearch Laboratories (West Grove, PA, United States). The Donkey F(ab’)2 anti-mouse IgG H&L (Alexa Fluor^®^ 568) for immunocytochemical staining was purchased from Abcam (cat # ab175699). DAPI fluoromount-G (cat # 0100-20) for mounting slides was purchased from Southern Biotech (Birmingham, AL, United States). For immunoblot analysis, 5% Americanbio Inc., non-fat dry milk (cat # NC0115668; Thermo Fisher Scientific, Waltham, MA, United States) prepared in Tris-buffered saline with. 1% Tween-20 (TBS-T) was used to dilute all the primary antibodies, while the secondary antibodies were diluted directly in the TBS-T buffer.

### Quantitation of proteins by Western blotting

All animal experiments were carried out based on the National Institute of Health’s “Guide for the Care and Use of Animals” and as approved by the Florida International University’s Animal Care and Use Committee (IACUC). To quantify IQCK protein levels in the mouse brain, 12-month-old normal control C57BL/6 mice were euthanized by carbon dioxide overdose and decapitated immediately, and different brain regions such as the cortex (CX), hippocampus (HP), brainstem (BS) and cerebellum (CB), were rapidly dissected and separated on ice and placed into a 1% NP40 buffer (50 mM Tris-HCl (pH 8), 150 mM NaCl, 0.02% sodium azide, and 1% sodium Non-idet P-40) with a complete protease inhibitor mix supplemented with sodium vanadate and microcystin. Following uniform homogenization of all the samples, they were subjected to centrifugation at 14,000 rpm for 20 min at 4°C, and the lysate samples mixed with equal amounts of a loading buffer were loaded into each well and subjected to SDS-PAGE electrophoresis exactly as described previously (Lakshmana et al., 2009; Wang et al., 2013). The proteins were then transferred onto PVDF membranes, blocked with 5% milk prepared in 1% TBS-T buffer, and incubated overnight with primary antibodies followed by 1- to 2-h incubation with HRP-conjugated anti-mouse or anti-rabbit secondary antibodies. Protein signals were detected at different exposure times following incubation with Supersignal West Pico Chemiluminescent Substrate (cat # 34580; Thermo Fisher Scientific, United States). Quantification of Western blot signals was conducted using the freely available NIH ImageJ software. Actin signals were used to normalize protein levels in each sample.

### Immunocytochemical staining of cell lines

SH-SY5Y neuroblastoma cells, human microglia cell line HMC3, HeLa cells, and CHO cells were washed in cold phosphate-buffered saline (PBS), fixed in 4% paraformaldehyde (PFA) in PBS for 10 min, and washed thrice in Tris-buffered saline with. 1% Tween 20 detergent (TBST). Cell permeabilization was carried out using. 4% Triton X-100 for 5 min, followed by blocking with a blocking solution (normal goat serum, 1%; BSA, 3%; gelatin, 1%; Triton X-100, 0.2%; saponin 0.2%) for 30 min, incubation with the IQCK primary antibody overnight, incubation with the Alexa Fluor 568-conjugated anti-rabbit IgG secondary antibody for 1 h, and mounting with 4′,6-diamidino-2-phenylindole (DAPI)-containing Fluormount-G (SouthernBiotech, Birmingham, AL) to visualize the nuclei. Cells positively stained for IQCK and those negative for IQCK were captured in a BZX700 All-in-One microscopy system (Keyence Corp, Itaska, IL, United States) and analyzed.

### Immunohistochemical staining of IQ motif containing protein K in mouse model and brain sections of patients with Alzheimer’s disease

Following euthanasia with isoflurane, the mice were fixed intracardially in 4% PFA, the brains were cryoprotected in 30% sucrose in phosphate-buffered saline (PBS) for 3 days or until the brains were completely sunk and then frozen in a Tissue-Tek OCT compound. A 15-μm coronal brain section was cut in a cryostat (Leica) at −20°C. Similarly, normal control (NC) and AD brain sections were cut at 15-μm thickness. AD and NC brain tissues (hippocampus in all cases) were obtained from the “Harvard Brain Tissue Resource Center”, which is supported in part by PHS grant number R24MH068855. Tissue permeabilization was carried out using.4% Triton X-100 prepared in 1XPBS for 10 min. Non-specific binding was blocked by incubation in the blocking solution prepared as described above for 30 min. Primary antibodies such as anti-IQCK, anti-IBA1, anti-OLIG2, anti-GFAP, and anti-NeuN prepared in a blocking solution (1:100 dilution) were incubated overnight at 4^0^C with gentle shaking. Secondary antibodies such as Donkey F(ab’)2 anti-Mouse IgG H&L (Alexa Fluor^®^ 568; Abcam), Donkey F(ab’)2 anti-rabbit IgG H&L (Alexa Fluor^®^ 568; Abcam), and Donkey F(ab’)2 anti-Rabbit IgG H&L (Alexa Fluor^®^ 488; Abcam) at 1:500 dilution were incubated for 1 h at room temperature in the dark. The staining was followed by a final autofluorescence elimination step of incubation in an undiluted autofluorescence eliminator reagent (cat # 2160, EMD Millipore) for 1 min under vigorous shaking to prevent reagent precipitation. Finally, the slides were mounted with 4′,6-diamidino-2-phenylindole (DAPI) containing Fluormount-G (Southern Biotech, Birmingham, AL) to visualize nuclei, and fluorescence signals were imaged and captured in a BZX700 All-in-One microscopy system (Keyence Corp., Itaska, IL, United States).

### Statistical analysis

NIH’s ImageJ software was used to analyze and quantify immunolabeled proteins detected by Western blot analysis. All protein levels were normalized to β-actin levels to reflect true changes in each sample. All the statistical analyses were performed using the Instat3 software (GraphPad, San Diego, CA, United States). For comparisons of the levels of IQCK between two groups such as NC and AD, Student’s two-tail paired t-test was carried out. Data were presented as mean ± standard error of the mean (SEM) and were considered significant only if *p* < 0.05. ** indicates *p* < 0.01.

## Results

### SH-SY5Y and HeLa cells but not HMC3 microglial or CHO cells express IQ motif containing protein K

**FIGURE 1:**
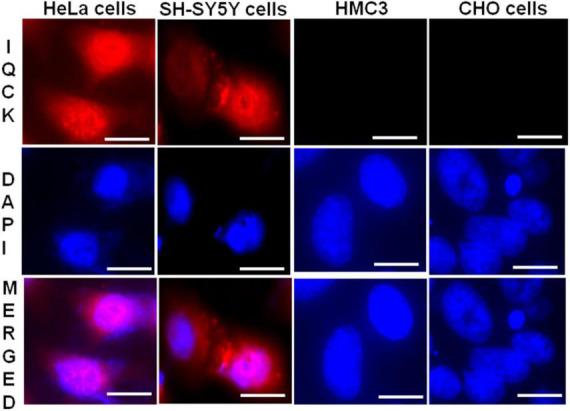
IQCK immunoreactivity is positive in SH-SY5Y and HeLa cells but negative in HMC3 microglial cells and CHO cells. Representative images are shown for different cell types with IQCK-immunoreactive (red) and DAPI-stained nuclei (blue). Merged images indicate IQCK expression in both the cytoplasm and nuclei. Scale bars indicate 20 μm.

The localization of a given protein in a cell dictates its function. As there is no information on IQCK subcellular localization and the type of cells expressing IQCK, we first analyzed the protein expression and cellular localization of IQCK in common cell lines by immunocytochemistry (ICC). The immunoreactivity with polyclonal IQCK-specific antibody clearly suggested IQCK expression in both SH-SY5Y and HeLa cell lines but not in HMC3 microglial cells or CHO cells (Figure 1). The high-resolution imaging analysis suggested that IQCK is expressed in both the nucleus and the cytoplasm. Since SH-SY5Y is a neuroblastoma cell line that can be differentiated into various functional neurons, IQCK immunoreactivity in these cells suggests that IQCK might be expressed in neurons. However, with no immunoreactivity observed in HMC3 cells that are derived from primary human microglia, the data imply that the IQCK protein may not be expressed in primary microglial cells. This negative result was also confirmed by a second IQCK polyclonal antibody. The positive staining in HeLa cells confirms IQCK expression in non-neuronal cells in addition to neuronal cells.

### IQ motif containing protein K protein is expressed in both mouse and human brain cells

**FIGURE 2:**
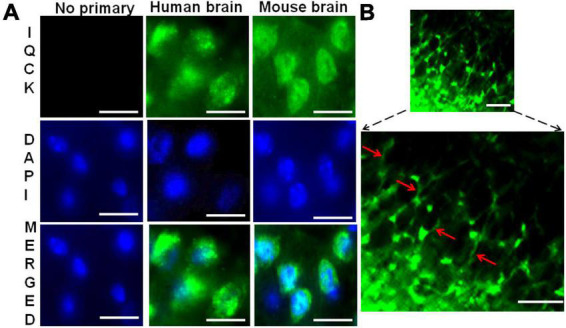
Immunohistochemical evidence for IQCK protein expression in both human and mouse brains. **(A)** Shows similar anti-IQCK antibody immunoreactive (green) cytoplasm and nuclei in human and mouse brain cells, while negative controls without the primary antibody lack signals. (B) Shows neuronal processes also expressing the IQCK protein. Scale bars indicate 20 μm in (A) and 50 μm in **(B)**.

To determine whether IQCK is expressed *in vivo* in the brain, we stained both mouse and human brain sections with an IQCK polyclonal antibody by immunohistochemistry (IHC). As seen in Figure 2A, similar to SH-SY5Y neuroblastoma cells, an intense IQCK immunoreactivity was detected in the cytoplasm as well as the nucleus in both mouse and human brain cells. Since negative controls lacking the IQCK primary antibody did not elicit any immunoreactivity, we concluded that the observed signals were specific to IQCK. Interestingly, we also observed IQCK protein expression in mouse brain neuronal processes (Figure 2B, lower panel). This validates that the IQCK protein is expressed in neurons. Thus, similar to *in vitro* results in cell lines, the IQCK protein is expressed *in vivo* in the brain.

### IQ motif containing protein K is robustly expressed in different regions of the mouse brain

**FIGURE 3:**
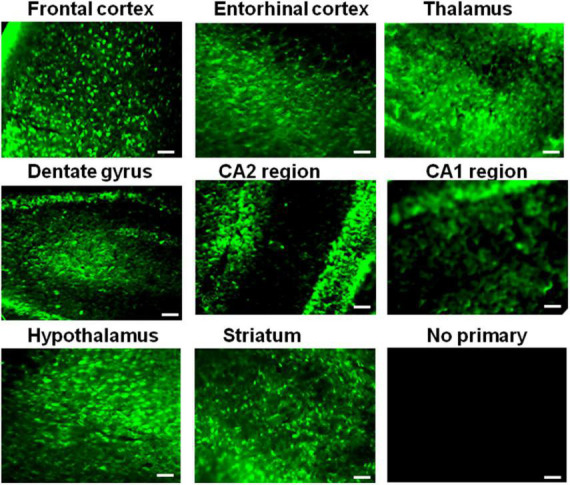
Different expression levels of the IQCK protein in mouse brain regions as revealed by immunohistochemistry. Representative micrographs are shown for different brain regions, as the images suggest differences in expression levels in the cortex and other brain regions. The lack of signals in the negative controls confirms the specificity of the used antibody for IQCK expression in the brain. Scale bars indicate 50 μm.

**FIGURE 4:**
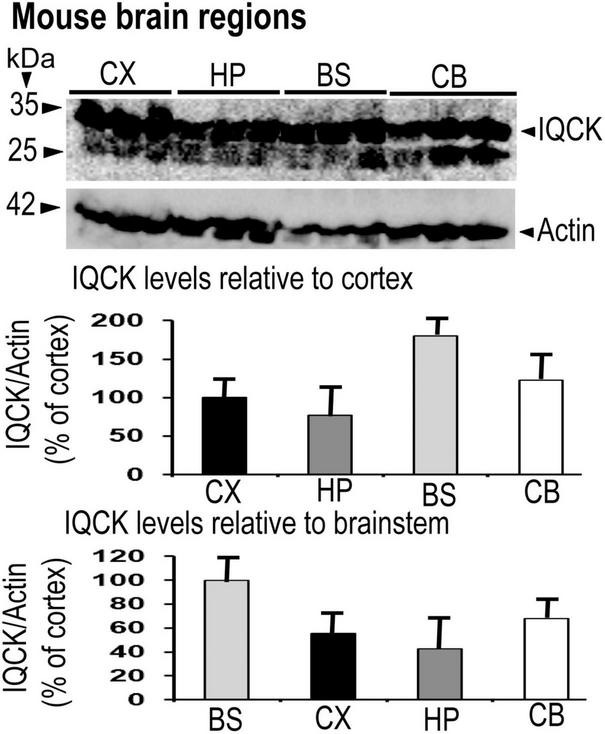
Demonstration of IQCK protein expression in different brain regions of 12-month-old mice by immunoblots. Mouse brain regions were lysed, and the lysates were subjected to SDS-PAGE electrophoresis. Protein levels of actin utilized as a loading control were used to normalize IQCK protein levels. Relative to the cortex (CX), IQCK expression levels in the hippocampus (HP) was 77%, in the brainstem (BS) was 180% and in the cerebellum (CB) was 123%. Relative to BS, the levels were 55% in the CX, 43% in the HP, and 68% in the CB.

Having confirmed IQCK protein expression in mouse and human brain tissues, we next assessed if there is any brain region-dependent IQCK expression given the unique structure and function of various brain regions. Therefore, we scanned multiple brain regions of the mice for IQCK regional immunoreactivity by IHC staining. The results suggest that cortical regions including the frontal and entorhinal cortices and subcortical regions such as the thalamus and striatum express IQCK robustly, while IQCK expression in hippocampal regions such as the dentate gyrus (DG), CA1, and CA2 is relatively low compared to that in the cortical regions (Figure 3). Also, in the cortical regions, IQCK expression appears to be seen in all layers. To confirm these results, with another method, we performed an immunoblot analysis of tissue lysates prepared from different regions of the mouse brain, and expression levels were normalized to those of β-actin protein levels used as a loading control. Consistent with IHC data in Figure 3, IQCK expression was higher in the cortex (CX) relative to the hippocampus (HP) which showed only 77% of CX, but the brainstem (BS) showed higher levels at 181% followed by the cerebellum (CB) which showed IQCK levels at 123% of CX (Figure 4). Relative to BS, CX showed 55%, HP showed 43%, and CB showed 67%. Thus, among the studied brain regions, BS showed the highest IQCK protein expression, while HP showed the lowest expression.

### IQ motif containing protein K is expressed in a cell-specific manner in the mouse brain

**FIGURE 5:**
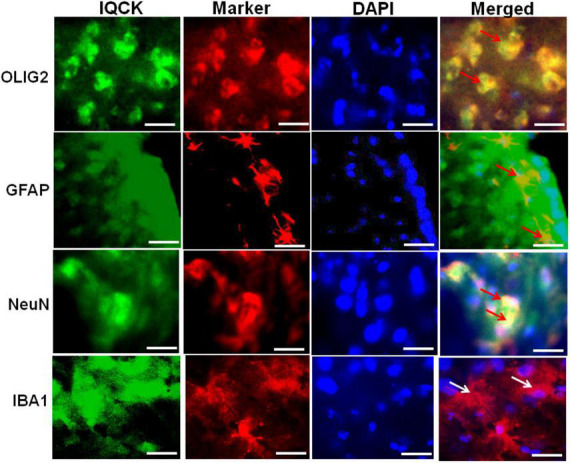
IQCK is expressed in different cell types in the cortical region of the mouse brain. Representative micrographs of cortical region serial sections that were immunostained with cell-type-specific antibodies such as Iba1 for microglia, NeuN for neurons, Gfap for astrocytes, and Olig2 for oligodendroglia (shown in red fluorescence) together with the polyclonal IQCK antibody (green fluorescence), and sections were counterstained with DAPI for detecting nuclei (blue fluorescence). IQCK expression was present in neurons, oligodendrocytes, and astrocytes but not in microglia. Control slides without the primary antibodies showed no signals (data not shown). The yellow color in the merged images demonstrates IQCK expression in the specific cell types as indicated by red arrows. White arrows for microglia indicate negativity for IQCK expression. Scale bars in all the images represent 50 μm.

Since the brain has multiple cell types with both unique and inter-dependent functions among them, we analyzed whether the IQCK protein is expressed in any or all types of brain cells. We used previously validated cell-specific markers such as Iba1 for microglia (Ito et al., 1998), NeuN for neurons (Mullen et al., 1992), Gfap for astrocytes (Eng, 1985), and Olig2 for oligodendrocytes (Yokoo et al., 2004). Co-immunostaining using a polyclonal IQCK antibody and cell-specific monoclonal antibodies showed IQCK expression in neurons, astrocytes, and oligodendrocytes but not in microglia (Figure 5). Thus, the yellow color seen in merged images after staining with both IQCK and cell-specific antibodies suggests co-expression of the antigens. Brain sections incubated with only secondary antibodies showed no signals (data not shown), suggesting that the observed signals are specific to antigens. The expected morphological features of cells stained with cell-specific antibodies were clearly observed and thus validated the used antibodies. Although three cell types showed IQCK expression, it is important to note that not all cells of any given type expressed IQCK protein, including those of neurons. Importantly, we also found IQCK expression in neurons, which may have significant physiological implications. Neuronal expression is also supported by our finding that neuronal processes also showed positive signals, as shown in Figure 2. Thus, IQCK expression in specific cells of the brain may depend on physiological activity, developmental stage of a given cell type, and various other stress signals. Overall, based on our immunohistochemical data, we posit that IQCK expression may not be restricted to any one cell type in the brain. However, it should be noted that IQCK antibodies may cross-react with other IQ-motif-containing proteins that share amino acid homology with IQCK. Therefore, future studies should confirm the cell-specific expression of IQCK using multiple antibodies. Nonetheless, this is the first detailed immunohistochemical analysis of the cell-specific expression of IQCK in the mouse brain.

### IQ motif containing protein K protein levels are significantly increased in Alzheimer’s hippocampal region relative to normal controls

**FIGURE 6:**
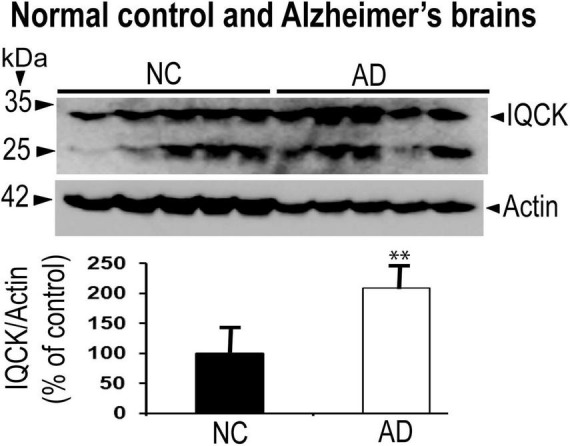
IQCK protein levels are significantly increased in the hippocampus of AD brains relative to NC brains by immunoblots. IQCK protein levels were normalized to actin levels used as loading controls, quantified by ImageJ, and compared between NC and AD. Relative to NC, the AD hippocampus showed 109% increase in IQCK levels. Data were statistically analyzed by paired *t*-test. **, *p* < 0.01; data are expressed mean + SEM, *n* = 5 per group.

**FIGURE 7:**
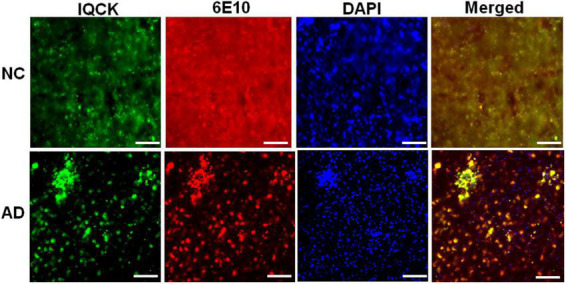
Immunohistochemical staining of NC and AD brain sections with polyclonal anti-IQCK and monoclonal 6E10 antibodies. Representative micrographs of the brain sections show increased IQCK-immunoreactive neurons (green) and 6E10 antibody-reactive Aβ and amyloid plaques (red) in the AD brains relative to the NC brain sections. Visualization of yellow color in the merged images indicates colocalization of the IQCK protein with Aβ and amyloid plaques. Scale bars in all the images represent 100 μm.

Given that multiple studies have considered IQCK as a risk gene for AD, we next investigated whether IQCK protein levels are altered in AD brains. To do this, we obtained clinically and pathologically confirmed AD hippocampal tissues and age-matched NC hippocampal tissues from the Harvard Brain Resource Centre and quantified the protein levels by Western blot analysis. The hippocampus is one of the earliest and most affected brain regions in AD and therefore is highly relevant. The demographics of control NC subjects and patients with AD are detailed in Table 1. The loading control, β-actin-normalized levels of IQCK was found to be markedly increased by 2-fold to 209% (**, *p* < 0.01, *n* = 5/group) in the AD hippocampus compared to the NC hippocampus (Figure 6). As expected, IHC staining confirmed the presence of anti-Aβ antibody (6E10)-positive amyloid plaques in the AD hippocampus (Figure 7, lower panels). Interestingly, the amyloid plaques were also positive for the IQCK antibody in co-staining experiments as reflected by the yellow color in the merged images in Figure 7 (lower panels), providing a theory that IQCK may be potentially involved in amyloid plaque development. It should also be noted that in plaque-free areas of the hippocampus, co-staining of Aβ and IQCK was also significantly associated with AD hippocampal tissues, but such co-staining was absent in the NC hippocampus. The increased IQCK staining in the AD hippocampus also validates the results from the immunoblot quantitation of IQCK protein levels, as shown in Figure 6. Thus, by both IHC and immunoblot analysis, our study provided the first evidence of increased IQCK protein levels in the AD hippocampus. Importantly, we also found an association between IQCK protein expression and amyloid plaques, implying a pathogenic role of IQCK in AD. In contrast, the NC hippocampal brain sections lacking detectable amyloid plaques demonstrated very light and diffused IQCK immunoreactivity (Figure 7, upper panel). Future studies should determine whether IQCK increases Aβ generation or simply inhibits Aβ degradation.

**TABLE 1:** Demographics of normal control and Alzheimer’s disease patients.

Subject #	Diagnosis	Age	Sex	PMI
NC1	Control	91	Female	30.1
NC2	Control	86	Male	25.28
NC3	Control	58	Female	26.6
NC4	Control	77	Female	28
NC5	Control	82	Male	24.08
AD1	Braak 6	95	Male	15
AD2	Braak 6	84	Male	6.66
AD3	Braak 6	72	Male	16.08
AD4	Braak 6	71	Male	16.62
AD5	Braak 5	87	Male	17.45

## Discussion

Although the IQCK gene has been consistently linked to increased risk of AD by multiple independent studies even after using stringent quality control protocols (Kunkle et al., 2019; Zhao et al., 2020; Chen et al., 2021), how IQCK may contribute to AD pathogenesis is completely unknown. Indeed, nothing is known about either the subcellular localization or tissue distribution or normal function of IQCK, especially in the brain. To understand both the normal function of IQCK and how IQCK may contribute to AD pathogenesis, as a first step, it is crucial to determine IQCK’s subcellular localization in cells, whether it is expressed in the brain, and, most importantly, what type of brain cells express or not express IQCK. Therefore, this study was initiated primarily to answer these questions. As there are no prior published studies on IQCK protein biology, the data presented in this study cannot be compared directly.

Our data on subcellular localization provide the first direct evidence of IQCK expression in both the nucleus and the cytoplasm in multiple cell types including neuroblastoma cells but not microglial cells. While the HMC3, SH-SY5Y, and HeLa cells that we analyzed here are human-derived, CHO cells are derived from Chinese hamsters, and therefore their negative reaction to the IQCK antibodies may be due to the specificity of the used IQCK antibodies on human and mouse tissues. Thus, whether CHO cells are truly negative for IQCK should be verified in future studies using other IQCK antibodies that are reactive to Hamster cells. Also, whether IQCK subcellular localization in human cells changes with altered physiological or pathological conditions is not yet known. In addition to cell lines, we demonstrated that the IQCK protein is expressed in both mouse and human brain sections, including the neuronal processes. Thus, our data suggest that the IQCK protein is localized not only to neuronal nuclei and the cytoplasm but also in neuritic processes *in vivo* in the brain. This might suggest a critical role of IQCK in brain function. Also, the IF staining revealed that the IQCK protein is widely distributed throughout the brain albeit with some differences in expression levels in each anatomical region. Our data showed that while the brainstem expressed the most, the hippocampus showed the lowest expression levels among the brain regions analyzed. This finding was also supported by our immunoblot data.

Another finding in this study is the cell type-specific expression of IQCK in the mouse brain by co-immunostainings utilizing well-authenticated markers of each cell type. Interestingly, consistent with data from microglial cell line HMC3, IQCK expression was absent in mouse brain microglial cells identified by co-staining with IBA1, a well-characterized marker of microglia (Ito et al., 1998). The cell shape stained by the IBA1 antibody is consistent with microglial morphology (Leyh et al., 2021). However, it should be noted that although IQCK was found to be expressed in neurons, astrocytes, and oligodendrocytes, not all cells of a given type expressed IQCK. For example, some neurons and oligodendrocytes did not show immunoreactivity to IQCK but others did. This may be because of the physiological state of the given cell or even because of subtypes in each cell type, which has as many as thirty distinct subtypes of neurons (Chen et al., 2017), and many subtypes of oligodendrocytes (Marques et al., 2016). Thus, IQCK expression in specific cell types might reflect its function in the brain.

The most important finding in this study is the significant increase in the protein levels of IQCK in AD brains by immunoblots, which was also supported by immunostaining. It is also important to note that the IQCK protein is present in amyloid plaques, suggesting that it may play a pathogenic role in amyloid plaque development or Aβ generation. Although we observed a predicted molecular mass of 34 kDa for the IQCK protein by Western blots in both mouse and human brains, it should be noted that we also observed another band of about 25 kDa. At this point, it is not clear whether this 25-kDa protein is an isoform or a cleaved product of full-length IQCK protein. Additionally, we found another stronger band at about 55 kDa. Initially, we used the IQCK polyclonal antibody from Proteintech and detected the 55 kDa protein band and a faint band at about 34 kDa. To test further, we next used another IQCK polyclonal antibody from BIOSSUSA and clearly detected the expected size of 34 kDa in the human brains and an additional band at 25 kDa. We tested two additional polyclonal antibodies from Novus Biologicals and AVIVA Systems Biology. Among the antibodies tested, the BIOSSUSA polyclonal antibody detected a clear band at about 34 kDa in the NC and AD brains. As of now, there are no monoclonal antibodies available for IQCK, and future studies should confirm these findings using specific monoclonal antibodies with minimal cross-reactivity.

IQ motif-containing proteins are widely distributed in nature, are well-known to bind to calmodulin, and influence calcium signaling, thereby regulating a wide variety of physiological functions (Bähler and Rhoads, 2002; Guo et al., 2021). The calcium/calmodulin-dependent protein kinase II (CaMKII) is a key protein kinase that plays a crucial role in neural plasticity and memory (Zalcman et al., 2018). Future studies should address whether IQCK also binds to calmodulin and plays any role in memory or neural plasticity in general.

## Data availability statement

The original contributions presented in this study are included in the article/supplementary material, further inquiries can be directed to the corresponding author.

## Ethics statement

The animal study was reviewed and approved by the Institutional Animal Care and Use Committee of Florida International University.

## Author contributions

HW: methodology, validation, formal analysis, investigation, data curation, writing – review and editing, and visualization. DD: methodology, validation, formal analysis, investigation, and visualization. MN: conceptualization, formal analysis, resources, writing – review and editing. HC: validation, formal analysis, resources, and writing – review and editing. ML: conceptualization, writing – review and editing, supervision, data curation, project administration, and funding acquisition. All authors agreed to be accountable for the content of the article.

## Conflict of interest

The authors declare that the research was conducted in the absence of any commercial or financial relationships that could be construed as a potential conflict of interest.

## Publisher’s note

All claims expressed in this article are solely those of the authors and do not necessarily represent those of their affiliated organizations, or those of the publisher, the editors and the reviewers. Any product that may be evaluated in this article, or claim that may be made by its manufacturer, is not guaranteed or endorsed by the publisher.
